# Alpha–fetoprotein elevation in NUT midline carcinoma: a case report

**DOI:** 10.1186/s12885-017-3262-0

**Published:** 2017-04-13

**Authors:** Lorenzo D’Ambrosio, Erica Palesandro, Marina Moretti, Giuseppe Pelosi, Alessandra Fabbri, Fabrizio Carnevale Schianca, Massimo Aglietta, Giovanni Grignani

**Affiliations:** 1 Division of Medical Oncology, Candiolo Cancer Institute – FPO, IRCCS, Strada Provinciale 142 Km 3.95, 10060 Candiolo, TO Italy; 2 Department of Oncology, University of Torino Medical School, Strada Provinciale 142 Km 3.95, 10060 Candiolo, TO Italy; 3 Department of Oncohematology, University of Perugia, Santa Maria Hospital, Via Tristano di Joannuccio 1, 05100 Terni, Italy; 4 Department of Pathology and Laboratory Medicine, Fondazione IRCCS Istituto Nazionale dei Tumori, Via Venezian 1, 20133 Milan, Italy; 5 Department of Oncology and Hemato–Oncology, University of Milan, Via Venezian 1, 20133 Milan, Italy

**Keywords:** NUT midline carcinoma, Mediastinal mass, Alpha–fetoprotein, Case report

## Abstract

**Background:**

Nuclear protein in testis (NUT) midline carcinoma is a rarely diagnosed and potentially under-recognized type of squamous carcinoma that is considered one of the most aggressive human solid tumors. Alpha-fetoprotein elevation has been associated with chronic liver diseases and a limited number of cancers. In particular, in presence of a mediastinal mass in a young man, alpha-fetoprotein elevation is considered nearly pathognomonic of a non-seminoma germ-cell tumor.

**Case presentation:**

A 22-year old man without any comorbidity was diagnosed with a large mediastinal mass with skeletal and lymph node metastases. The clinical picture was dominated by a life-threatening superior vena cava syndrome with elevated alpha-fetoprotein and lactate dehydrogenase that supported the diagnostic suspicion of mediastinal germ-cell tumor. However, a biopsy showed a poorly-differentiated and diffusely necrotic carcinoma. We eventually reached the diagnosis of the peculiar entity of NUT midline carcinoma, but the differential diagnosis was quite challenging also because alpha-fetoprotein is not reported as a marker of NUT midline carcinoma. Notably, alpha-fetoprotein levels correlated with disease course.

**Conclusions:**

The life-threatening aggressiveness of NUT midline carcinoma mandates to reach the right diagnosis in the shortest possible time. In this regard, poorly differentiated carcinomas lacking glandular differentiation mandate testing for NUT expression by immunohistochemistry. Awareness of a potentially misleading tumor marker elevation can help to broaden the differential diagnosis and establish the most appropriate treatment.

## Background

Nuclear protein in testis (NUT) midline carcinoma is an exceedingly rare subtype of squamous poorly–differentiated carcinoma and is considered one of the most aggressive human solid tumors [1–3]. NUT midline carcinoma is a relatively new entity, that may likely be under–recognized and under–diagnosed. All ages and organs might be affected, but most frequently NUT midline carcinoma arises along the trunk or the head and, in particular, in midline structures such as the mediastinum [1–3]. NUT midline carcinoma is characterized by the pathognomonic chromosomal rearrangement between the NUT gene with either bromodomain–containing protein 4 (BRD4) or, less frequently, with BRD3 (on chromosome 9), leading to the fusion genes BRD4–NUT or BRD3–NUT, respectively [1–3]. BRD is a DNA reader that activates transcription by binding to acetyl–modified lysine residues of histone tails [1, 3, 4]. The expression of several oncogenes, including transcription factors and *MYC*, is epigenetically regulated by BRD. More recently, a novel fusion gene between the methyl-transferase protein NSD3 on chromosome 8 and NUT has been reported (NSD3–NUT) [1, 5, 6]. NSD3 has shown to be associated with the extraterminal domains of BET proteins, which serves as a key component in the BRD–NUT oncogene complex [1]. Moreover, two novel three–way translocations [t(9;15;19;q34;q13;p13.1), and t(4;15;19)(q13;q14;p13.1)] have been described [5, 6]. NUT rearrangement has been suggested to possibily represent a tumor–initiating event. Its importance in the pathogenesis of NUT midline carcinoma is further supported by the evidences that withdrawal of the NUT fusion protein resulted in a dramatic and irreversible squamous differentiation and growth arrest, thus demonstrating that the BRD–NUT protein blocks differentiation [7].

Alpha–fetoprotein elevation has been associated with chronic liver diseases and a limited number of cancers (hepatic primary tumors and metastases, bile duct, pancreatic, gastric and lung cancer, and non–seminoma germ–cell tumors). Moreover, in presence of testicular or mediastinal mass an elevated alpha–fetoprotein strongly suggests a non–seminoma germ–cell tumor especially in young patients.

## Case presentation

**Fig. 1:**
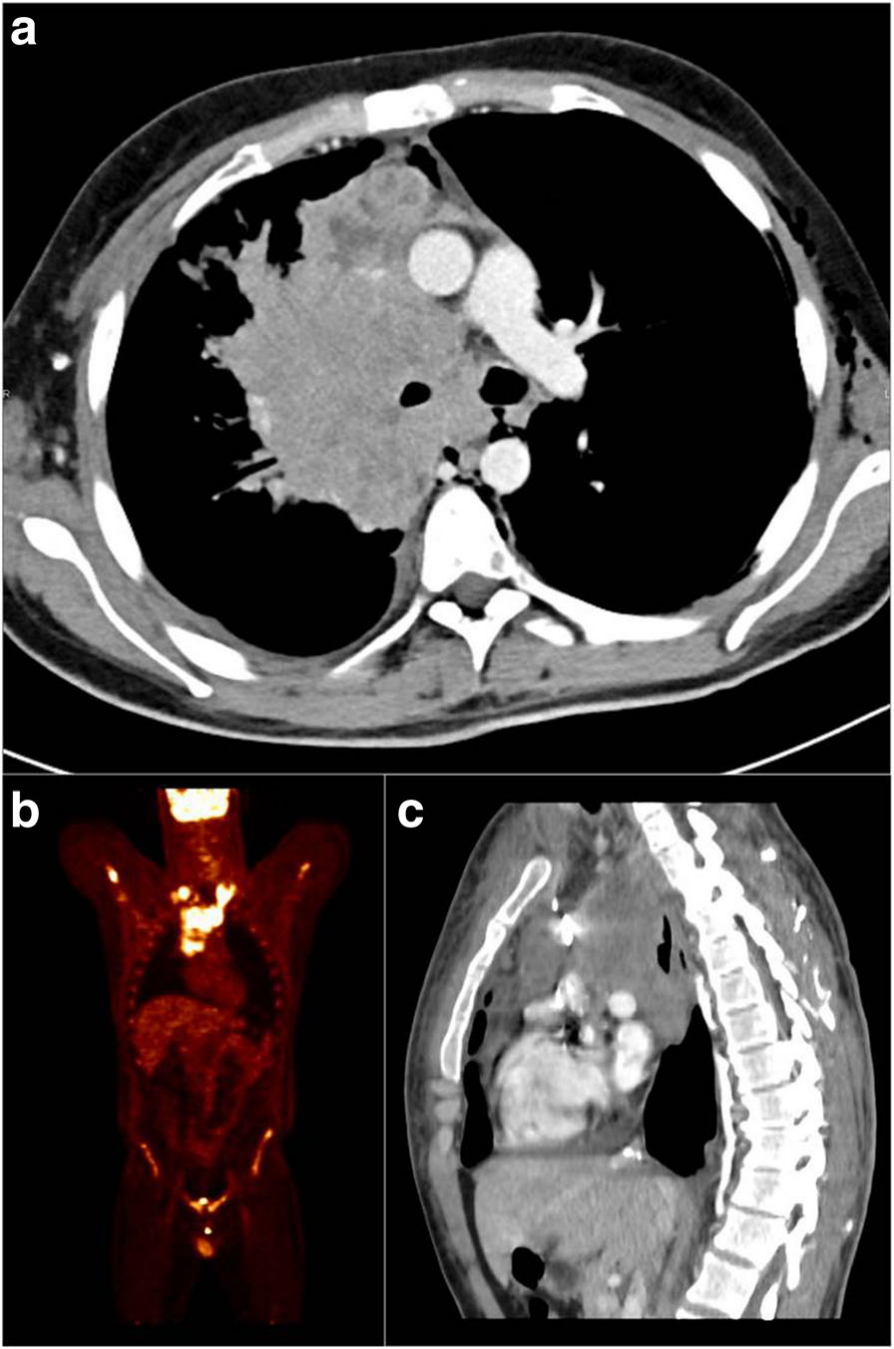
Disease status at diagnosis. Panel **a**, **c**: CT scan showing bulky mediastinal mass with involvemente of right lung ilum, compression of the right superior bronchus, and dislocation of the trachea and right intermediate bronchus with pleural and pericardial effusion (panel **a** axial, panel **c** sagittal). Panel **b**: PET scan showing supraclavicular involvement of the disease and absence of liver metastases. CT, computed tomography; PET positron emission tomography

**Fig. 2:**
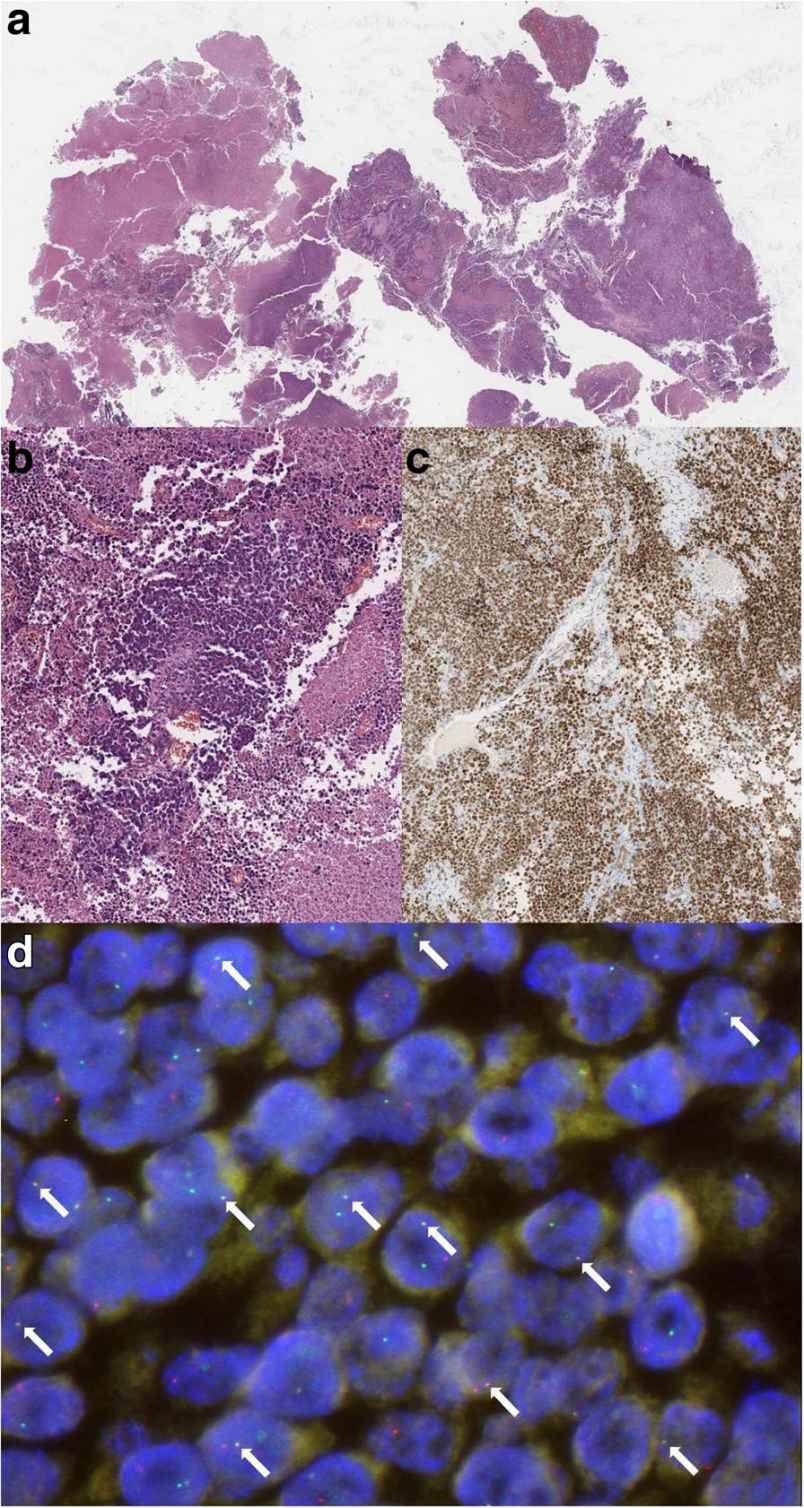
Pathology images. Panel **a**, **b**: Hematoxilin and eosin stain (panel **a** 20X magnification, panel **b** 40X magnification). NUT carcinoma typically shows sheets of monomorphic round to ovoid cells with scant pale eosinophilic to basophilic cytoplasm; nuclei with irregular outlines and slightly coarse chromatin with small nucleoli. Necrosis, mitotic figures and crush artefact are common. Panel **c**: diffuse nuclear immunohistochemical staining with nuclear protein in testis (NUT) antibody (40X magnification). NUT (C52B1, CELL Signaling, Technology) Rabbit mAb detects endogenous levels of total NUT protein. The antibody also detects levels of BRD4-Nut fusion protein. Panel **d**: FISH showing positivity for NUT-BRD4 fusion gene. FISH was performed using dual color single fusion probes: BAC clone (RP11-122P18) spanning NUT gene is labeled in spectrum orange, BAC clone RP11-637P24 covering BRD4 gene is labeled in spectrum green. The presence of the translocation t (15;19)(q14;p13) BRD4/NUT is showed by overlapping of one green and one orange probe signal (white arrows). FISH, fluorescence in situ hybridization; NUT, nuclear protein in testis; BRD4, bromodomain–containing protein–4; BAC, bacterial artificial chromosome

A 22–year old man was referred to our center because of a large mediastinal mass along with skeletal and lymph node metastases. The clinical picture was dominated by shortness of breath due to severe superior vena cava syndrome with pleural–pericardial effusion (Fig. 1 panels a, b, c). Laboratory tests showed elevated alpha–fetoprotein (765 ng/mL; normal value <10.9 ng/mL) and lactate dehydrogenase (LDH) = 14,468 U/L (normal range 240–480 U/L). Taken altogether these findings strongly suggested a mediastinal non–seminoma germ cell tumor (GCT) [7] but, unexpectedly, a biopsy of a supraclavicular enlarged lymph node revealed a poorly differentiated and diffusely necrotic carcinoma (Fig. 2, panels a, b). Immunohistochemistry was weakly positive for AE1–AE3 cytokeratin pool, TTF–1, p63, synaptophysin, chromogranin A, CD34 and, focally, EMA and cytokeratin 7, but negative for PLAP, alpha–fetoprotein, beta–HCG, CD30, CD3, CD20, CD117, Melan–A, S100 protein, ALK, PAX8, and desmin, thereby ruling out GCT (Table.1 describes detailed immunohistochemistry profile and clues to diagnosis suggested by each immunohistochemical marker) [7]. Due to such peculiar presentation and the high levels of alpha–fetoprotein, a second pathology opinion was requested. GCT was again excluded and a diagnosis of poorly differentiated large cell carcinoma with neuroendocrine differentiation was rendered. However, taking advantage of the clinical presentation and the patient’s young age, in a third consultation, a monoclonal antibody against NUT protein was used (Fig. 2, panel c) [8] along with fluorescence in situ hybridization (FISH) analysis for NUT–BRD4 translocation [3]. Tumor cells revealed diffuse decoration for NUT protein and FISH analysis confirmed the occurrence of t(15;19) BRD4–NUT translocation (Fig. 2 panel d), thereby establishing the diagnosis of NUT midline carcinoma [1, 3].

**Table 1:** Immunohistochemistry profile at the time of first evaluation

Weakly positive	Clues to diagnosis (if positive)	Negative	Clues to diagnosis (if positive)
AE1-AE3 cytokeratin pool	Carcinomas, GCT (rare), thymoma, mesenchymal tumors	PLAP	Seminoma
TTF-1	Lung, thyroid cancer	AFP	Non-seminoma GCT, HCC, pancreas carcinoma
p63	Basal/squamous carcinoma, mesenchymal tumors, lymphomas, adenoid cystic carcinoma, salivary gland tumors, thymoma, thymic carcinoma, breast, urothelial carcinoma	Beta-HCG	Non-seminoma GCT
Synaptophysin	Neuroendocrine neoplasms, adrenal gland carcinoma, mesenchymal tumors	CD30	Lymphomas (HL and NHL), non-seminoma GCT,
Chromogranin A	Neuroendocrine tumors	CD3	T-cell lymphomas
CD34	Hematologic malignances, mesenchymal tumors, papillary thyroid carcinoma, thymoma	CD20	B-cell lymphomas
Focally positive		CD117	Mesenchymal tumors, GCT, SCLC, leukemias, lymphomas, melanoma
EMA	adenocarcinoma, renal cell carcinoma, choriocarcinoma, mesenchymal tumors, mesothelioma, lymphoma, thymic carcinoma	Melan-A	Melanoma, mesenchymal tumors, adrenal cortical tumors, sex-cord stromal tumors
Cytokeratin 7	Lung cancer and other carcinomas, GCT (rare), mesenchymal tumors	S-100	Melanoma, mesenchymal tumors, adenoid cystic carcinoma, sex-cord stromal tumors
		ALK	Lymphomas, NSCLC, mesenchymal tumors, RCC (rare)
		PAX8	Thymic carcinoma, thymomas, thyroid anaplastic carcinoma, RCC, neuroendocrine neoplasms, seminoma
		Desmin	Mesenchymal tumors

*TTF-1* thyroid transcription factor-1, *EMA* epithelial membrane antigen, *PLAP* placental alkaline phosphatase, *AFP* alpha-fetoprotein, *beta-HCG* human chorionic gonadotropin, *ALK* anaplastic lymphoma kinase, *GCT* germ cell tumor, *HCC* hepatocellular carcinoma, *HL* Hodgkin’s lymphoma, *NHL* Non-Hodgkin lymphoma, *RCC* renal cell carcinoma, *SCLC* small cell lung cancer, *NSCLC* non-small cell lung cancer

**Fig. 3:**
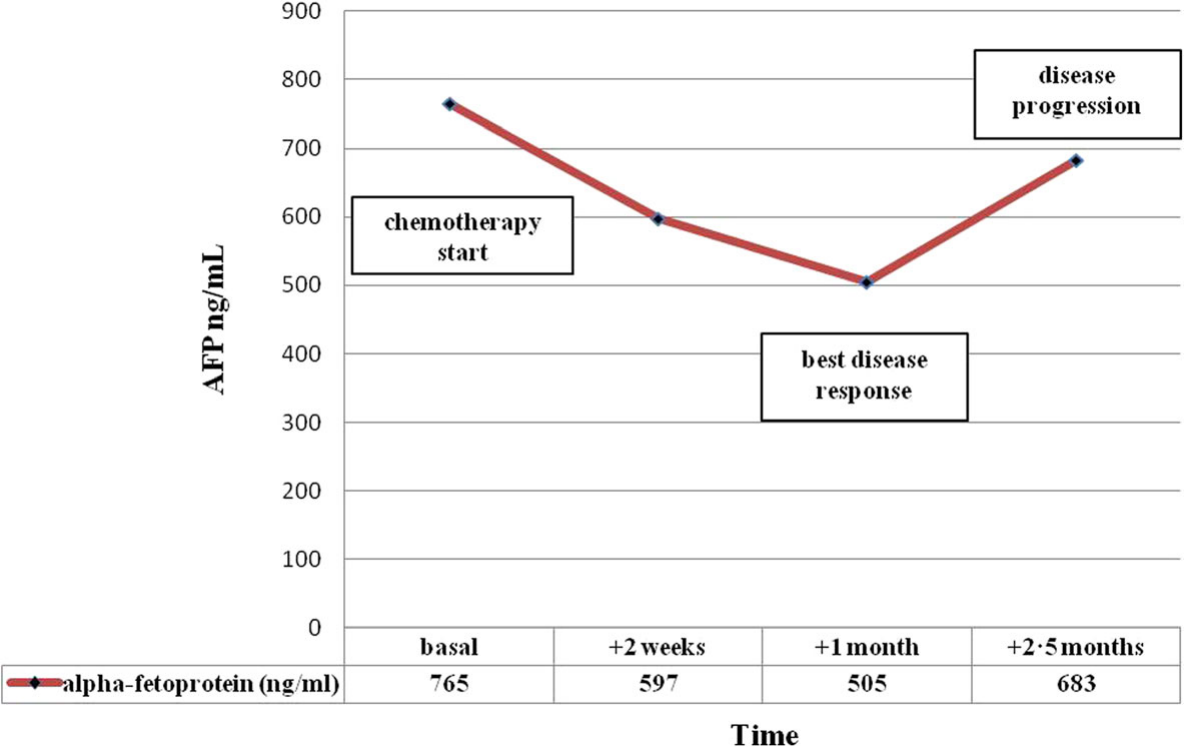
Alpha-fetoprotein levels and disease course during chemotherapy

Notably, in our patient alpha–fetoprotein levels directly correlated with disease course. Indeed, in this young gentleman, basal level was 765 u/mL and declined to 505 u/mL after two chemotherapy cycles (cisplatin 25 mg/m2 days 1- > 4, etoposide 100 mg/m2 days 1- > 4) along with a sharp clinical improvement due to attenuation of the superior vena cava syndrome. Unfortunately, clinical benefit was short–lasting and alpha–fetoprotein rose up again at the time of subsequent disease progression (Fig. 3).

## Discussion and conclusions

Diagnosis of NUT midline carcinoma is quite challenging and demands to be hypothesized and specifically demonstrated. Indeed, microscopic features are quite unspecific and range from completely undifferentiated carcinoma to carcinoma with squamous differentiation and abrupt keratinization that may be either focal or prominent. Tumor cells usually have an epithelioid, medium–size appearance and are in general monomorphic with round to oval nuclei and distinct nucleoli. Further morphologic aspects are a high mitotic rate along with necrosis and neutrophilic infiltrates. Immunohistochemistry with the NUT antibody is positive with a diffuse nuclear staining. However, NUT positivity might be found in GCTs as well and, to be regarded as truly positive, it must be seen in >50% of tumor cells.

Another peculiarity of NUT midline carcinoma is represented by the relatively simple karyotype that often presents the NUT translocation as the only relevant abnormality. This strongly differentiate this tumor from other squamous cell carcinomas that usually harbor highly aberrant and complex karyotypes [9, 10].

Differential diagnosis includes several tumors, encompassing hematologic malignancies or neuroendocrine tumors, because of non–specific morphology and immunohistochemistry profile apart from the pathognomonic NUT protein expression and the characteristic cytogenetic abnormalities [1, 3, 11]. As a general rule, it is recommended to perform immunohistochemistry for NUT protein in all poorly differentiated carcinomas that lack glandular differentiation and whenever the clinical picture is not completely consistent with more frequent tumors (midline or unusual position, young people, no smoking habit, rapid progression upon therapy) [2, 8, 12]. Both clinical history and some pathologic features (i.e., monomorphic tumor cells with abrupt keratinization) might help in generating the suspicion of NUT midline carcinoma. [10–12].

In the absence of hepatic involvement (Fig. 1 panel c), the diagnosis of NUT midline carcinoma was particularly challenging in our patient because alpha–fetoprotein has only seldom been reported associated with this tumor [1, 3, 13–16]. Table 2 describes other clinical cases reporting alpha–fetoprotein elevation. Moreover, in NUT midline carcinoma immunohistochemical expression of alpha-fetoprotein is negative, as it was in our patient and in other NUT midline carcinoma cases describing serum alpha-fetoprotein elevation [1, 3, 17]. Interestingly, alpha–fetoprotein levels during treatment were not reported in the majority of the cases, whereas this marker correlated with disease course in our patient. In light of these findings, we suggest not to exclude and, on the contrary, to take into consideration the diagnosis of NUT midline carcinoma when facing a patient with fast–growing lump in midline structures and alpha-fetoprotein elevation. Of course, evaluation of alpha–fetoprotein levels in other cases of NUT midline carcinoma might help to better define the role of this serum marker in this challenging disease.

**Table 2:** Case reports of NUT midline carcinoma describing alpha–fetoprotein (AFP) elevation

Authors	Year	Site(s) of disease	Age	Gender	Serum AFP level	IHC AFP
Zhu B et al.	2011	Mediastinum, lung	42	Male	Elevated	Negative
Ball A et al.	2013	Mediastinum, pelvis	19	Female	Elevated	NA
Parikh SA et al.	2013	Mediastinum, lung	36	Male	Elevated	NA
Raza A et al.	2015	Mediastinum, lung	36	Male	Elevated	Negative
Harada Y et al.	2016	Mediastinum, lung, bone	28	Male	Elevated	NA
Present report	2017	Mediastinum, lymphnodes, bone	22	Male	Elevated	Negative

*IHC* immunohistochemistry, *NA* not available

The observed elevation of alpha–fetoprotein in NUT midline carcinoma might be related to the suggested hypothesis that these cells arise from primitive neural crest–derived cells. [9] Indeed, both gene expression profile similar to adult ciliary ganglion [18] and the absence of the in-situ component are consistent with this cell of origin. [7].

Beyond its rarity, NUT midline carcinoma may initially and transiently respond to several cytotoxics [2] used to treat undifferentiated carcinomas, making even less likely the raising of the right diagnostic suspicion. Regardless the chosen treatment, activity was at most transient and short–lasting [2]. Therefore, to reach the correct diagnosis in the shortest possible time is crucial because prognosis is dismal, clinical conditions may worsen in few days, conventional chemotherapy is only marginally active and, finally, a new target therapy based on bromodomain inhibitors is showing promising results [4, 19, 20]. Despite being an experimental treatment, patients should be strongly encouraged to enroll in clinical trials to rapidly explore these innovative therapeutic strategies.

In conclusion, to avoid misleading diagnostic interpretation in case of alpha–fetoprotein elevation, sharing patient information between pathologists and clinicians assessing patients affected by poorly differentiated carcinoma arising nearby midline structures is crucial and we point out to consider NUT midline carcinoma in the differential diagnosis. Finally, in light of our observation, we also suggest to measure alpha–fetoprotein levels during tumor treatment to monitor disease course.

## Abbreviations

AFP: Alpha-fetoprotein
ALK: Anaplastic lymphoma kinase
beta-HCG: Human chorionic gonadotropin
BRD: Bromodomain-containing protein
EMA: Epithelial membrane antigen
FISH: Fluorescence in situ hybridization
GCT: Germ-cell tumor
LDH: Lactate dehydrogenase
NUT: Nuclear protein in testis
PLAP: Placental alkaline phosphatase
TTF-1: Thyroid transcription factor-1
